# Microbubbles as a contrast agent in grating interferometry mammography: an *ex vivo* proof-of-mechanism study

**DOI:** 10.1186/s41747-019-0097-2

**Published:** 2019-05-21

**Authors:** Kristina Lång, Carolina Arboleda, Serafino Forte, Zhentian Wang, Sven Prevrhal, Thomas Koehler, Norbert Kuhn, Bernd David, Konstantins Jefimovs, Rahel A. Kubik-Huch, Marco Stampanoni

**Affiliations:** 1 Swiss Light Source, ETH Zurich, Paul Scherrer Institute, 5232 Villigen, Switzerland; 2 Institute for Biomedical Engineering, University and ETH Zurich, Zurich, Switzerland; 3 Department of Radiology, Kantonsspital Baden, Im Ergel 1, 5404 Baden, Switzerland; 4 Philips GmbH Innovative Technologies, Research Laboratories, Philips Research Hamburg, Röntgenstrasse 24-26, 22335 Hamburg, Germany

**Keywords:** Contrast media, Interferometry, Mammography, Microbubbles, Phantoms (imaging)

## Abstract

Grating interferometry mammography (GIM) is an experimental breast imaging method at the edge of being clinically implemented. Besides attenuation, GIM can measure the refraction and scattering of x-rays resulting in differential phase contrast (DPC) and dark-field (DF) images. In this exploratory study, we assessed the feasibility of using microbubbles as a contrast agent in GIM. Two millilitres of microbubbles and iodine were respectively injected into *ex vivo* breast phantoms, consisting of fresh chicken breasts. Native and postcontrast images were acquired with a clinically compatible GIM setup, operated at 38 kVp, 14-s acquisition time, and with a dose of 1.3 mGy. The visibility of the contrast agents was analysed in a side-by-side comparison by three radiologists. The contrast-to-noise-ratio (CNR) was calculated for each contrast agent. We found that both contrast agents were judged to be visible by the readers. The mean CNR was 3.1 ± 1.9 for microbubbles in DF and 24.2 ± 6.5 for iodine in attenuation. In conclusion, this is a first proof-of-mechanism study that microbubbles could be used as a contrast agent in clinically compatible GIM, due to their scattering properties, which implies the potential use of a contrast agent with a high safety profile in x-ray-based breast imaging.

## Key points

- Grating interferometry mammography is a novel breast imaging technique at the edge of being clinically implemented
- Grating interferometry mammography obtains attenuation, phase-contrast, and dark-field images in one acquisition
- Microbubbles scatter x-rays, which has yet to be demonstrated in a clinical setup
- Microbubbles were shown to give contrast enhancement in the dark-field mode
- Microbubbles could potentially be used as a contrast agent in clinically compatible grating interferometry mammography

## Background

In conventional x-ray imaging, the attenuation of x-rays is used to create an image. However, x-rays are not only attenuated by matter but further refracted and scattered. So far, these signals have not been considered in medical imaging, but have recently been the focus of the developing research field of phase-contrast imaging [1, 2]. With the advent of the x-ray grating interferometry technique, the refraction and the small-angle and ultra-small angle scattering signals can be measured resulting in differential phase contrast (DPC) and dark-field (DF) images, in addition to the attenuation image.

The grating interferometry approach has enabled the translation of phase-contrast imaging to a clinical setting, since it works with a conventional x-ray tube and detector [3]. This emerging technique has been of special interest in the breast imaging field, with several potential clinical benefits [2, 4–7]. DPC provides edge enhancement which can improve soft tissue contrast [8], and the DF signal can be used to characterise tissues based on their scattering properties. As an example of the latter, it has been shown that the DF signal has the potential to discriminate between different types of microcalcifications and could enable the opportunity to non-invasively distinguish between microcalcifications associated with benign lesions and those associated with malignant lesions, which in turn can become an important clinical tool to reduce false positives and biopsy rates [9–11]. However, these studies have been performed in a preclinical setting and further evaluation in a clinical context is required.

Furthermore, the additional imaging signals with GIM could also open up new possibilities for the use of contrast agents. In breast imaging, there are currently several modalities that gain increased diagnostic performance by adding contrast agents to visualise tumour angiogenesis, such as gadolinium-enhanced breast magnetic resonance imaging (MRI), iodine-enhanced spectral mammography, and contrast-enhanced ultrasound using microbubbles [12, 13]. However, the safety profiles of these contrast agents differ. In recent years, concerns have been raised on the potential harm of using gadolinium with reports showing gadolinium deposits in the brain after repeated contrast-enhanced MRI examinations [14, 15]. The potential morbidity, if any, of these deposits has not yet been established but is currently being investigated. With respect to x-ray contrast agents, it is well known that iodinated contrast agents can lead to adverse events, ranging from allergic reactions to contrast-induced nephropathy [16]. Microbubbles, on the other hand, constitute a contrast agent with a high safety profile that is used in ultrasound imaging, especially applied to cardiac, liver, and breast imaging [17, 18]. The bubbles create contrast by reflecting the ultrasound waves but also inhere the possibility to scatter x-rays and could therefore potentially be used as a contrast agent in GIM. Accordingly, microbubbles have been shown to give detectable contrast using phase-contrast imaging with synchrotron-radiation imaging [19–21] and with a preclinical grating-interferometry x-ray system [22], but have not previously been shown using a clinically compatible grating-interferometry x-ray system.

The purpose of this *ex vivo* study was to explore if microbubbles could be used as a contrast agent in clinically compatible GIM.

## Methods

In this exploratory proof-of-mechanism study, we used *ex vivo* phantoms consisting of four fresh chicken breasts (5 × 5 × 3 cm). The chicken tissue is dense and could therefore be considered to represent a dense breast. Fragments of eggshells of approximately 0.5–1 mm were placed on top of the chicken simulating a calcification cluster and to be used as a positive contrast reference. Two different commercially available contrast agents were employed: a microbubble ultrasound contrast agent (SonoVue 8 μl/ml, Bracco, Milan, Italy) and an iodinated contrast agent (Iopamiro 370 mg/ml, Bracco, Milan, Italy). The mean diameter of SonoVue’s microbubbles is 2.5 μm, and more than 90% of the bubbles are smaller than 8 μm [23]. Two millilitres of undiluted microbubbles and iodine were respectively injected directly into the phantoms at two different locations. Native and post-contrast images were obtained.

### Image acquisition

**Fig. 1:**
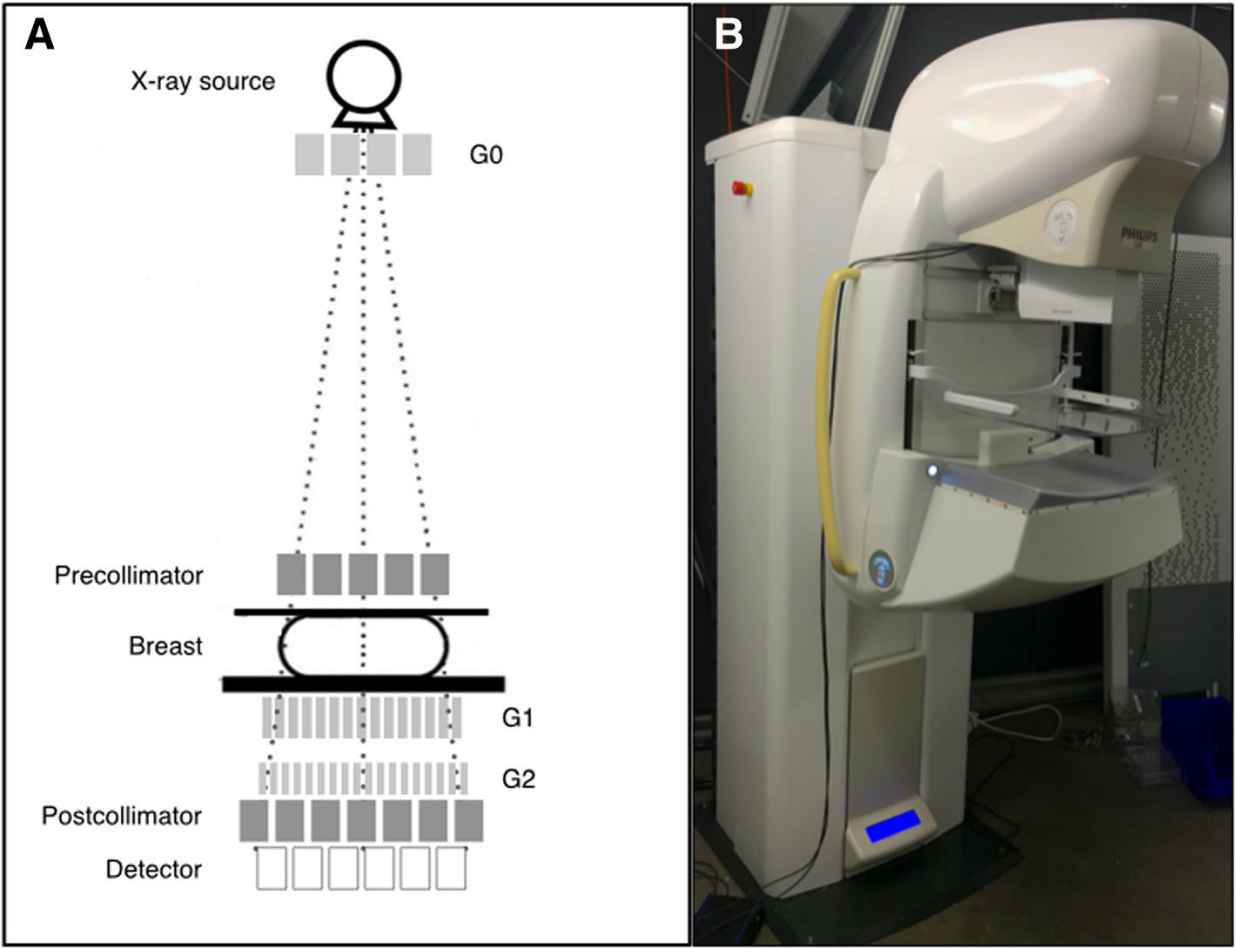
**a** Sketch of the grating interferometer installed in a Philips MicroDose mammography system. The gratings are thin diffraction elements placed in the beam perpendicular to the optical axis, containing periodical line structures that absorb x-rays and/or shift their phase. A source grating (G0), a beam splitter grating (G1), and an analyser grating (G2) contribute at different steps to the recording of the phase and dark-field signals. **b** Photo of the grating interferometry mammography investigational device used in the study

Images were acquired using a clinically compatible GIM prototype [2, 24]. The system was based on a Philips MicroDose Mammography system (Philips Health Systems, Kista, Sweden) that had been modified with the insertion of a grating interferometer (Fig. 1). The attenuation and DPC and DF signals were extracted from the measured interference pattern, thanks to a slit-scanning phase retrieval algorithm [24]. These signals were reconstructed through an iterative method [24] and post-processed with band removal and median-filtering to remove noise. The system was operated at 38 kVp with an acquisition time of 14 s. The total examination time, including handling of the sample, was estimated to roughly 7 min. The air Kerma, measured with a RaySafe X2 device (Unfors RaySafe AB, Billdal, Sweden), was 4 mGy, which translates into a mean glandular dose of 1.3 mGy, taking into account the phantom thickness and assuming a glandular density of 100% [25].

### Contrast assessment

The visibility of the contrast agent in the attenuation and DF mode was analysed in a side-by-side comparison by three experienced (> 5 years) breast radiologists in consensus. In addition, the contrast-to-noise-ratio (CNR) for microbubbles and iodine was calculated. Region-of-interests (ROIs) were placed adjacent to the needle tip to include the visible distribution of the bubbles, and in the centre of the iodine distribution. The following formula was used to calculate the CNR for each pixel of the contrast agent ROI:

1 $$ {\mathrm{CNR}}_{\mathrm{pixel}}=\left(\frac{\left|{I}_{\mathrm{CA}}-{\mu}_{\mathrm{tissue}}\right|}{\sigma_{\mathrm{tissue}}}\right) $$

where *I*_CA_ corresponds to the pixel-wise intensity of the contrast agent ROI, while *μ*_tissue_ and *σ*_tissue_ are the mean and standard deviation of the tissue ROI, respectively. Afterwards, the mean and standard deviation of CNR_pixel_ were calculated.

## Results

**Fig. 2:**
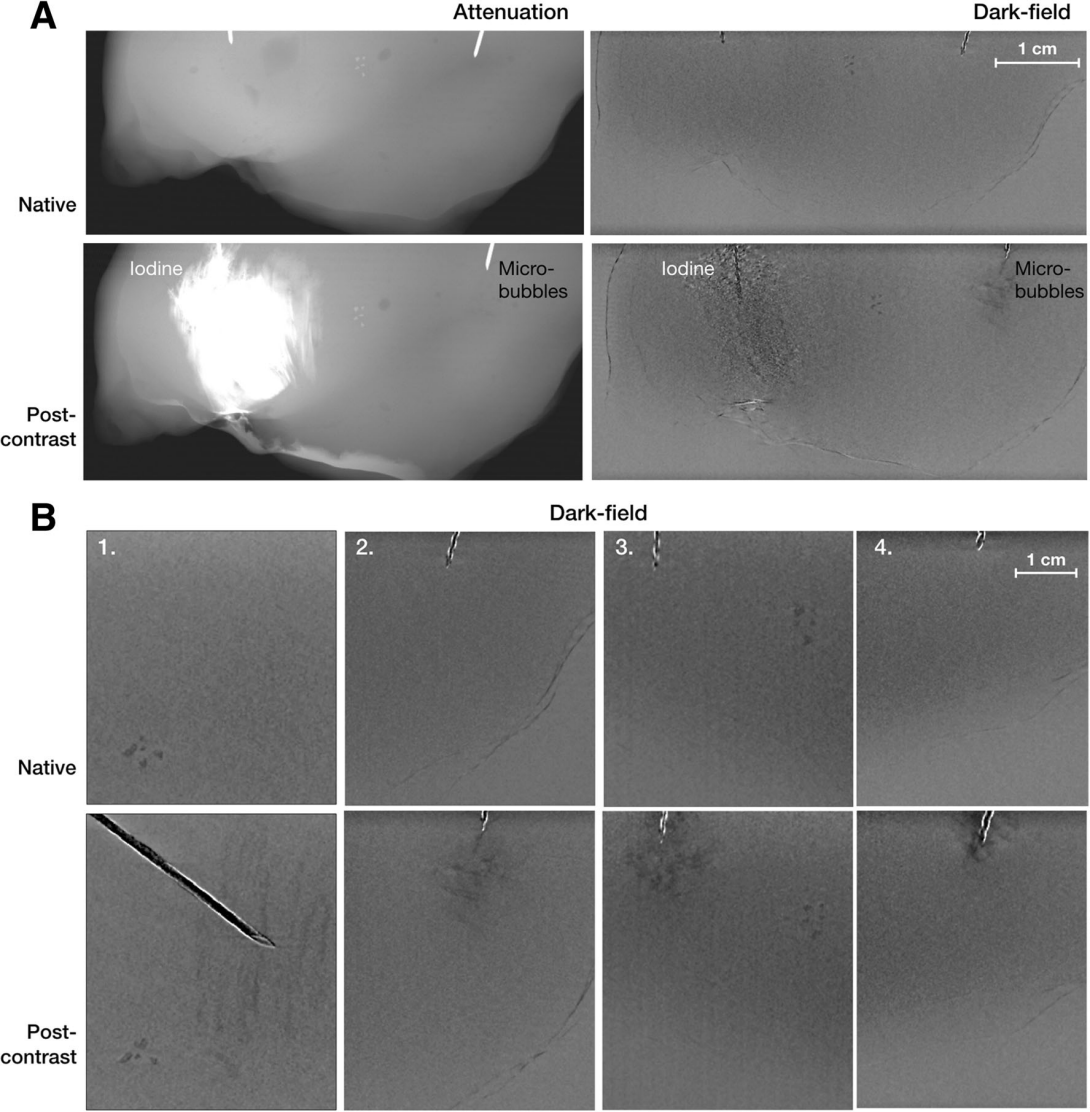
**a** Native and post-contrast grating interferometer mammograms of the chicken breast injected with iodine and microbubbles. The iodine is visible in both the attenuation image (left) and in the dark-field image (right), whereas the microbubbles are only visible in the dark-field mode. The egg-shells (centre of the sample) were visible in both the attenuation and the dark-field image. **b** A display of the dark-field images of all four samples before and after the injection of microbubbles

The radiologists judged the microbubbles to be visible in the DF image in all four samples. The microbubbles were not visible in the attenuation image. The iodine was visible both in the attenuation and DF images, but with a much higher contrast in the former (Fig. 2a). The mean CNR was 3.1 ± 1.9 for microbubbles in the DF mode and 24.2 ± 6.5 for iodine in attenuation mode (Table 1). Even if the microbubbles had a lower CNR in the DF image compared to the iodine in the attenuation mode, the background in the DF image was homogeneous making the microbubbles stand out (Fig. 2b).

**Table 1:** Contrast-to-noise-ratio of the contrast agents calculated in both the attenuation and dark-field modes

Sample number	Attenuation		Dark-field	
	Iodine	Microbubbles	Iodine	Microbubbles
1	9.7 ± 2.1	1.4 ± 0.3	3.8 ± 2.4	2.7 ± 1.3
2	46.0 ± 11.7	1.7 ± 0.7	5.1 ± 2.8	2.9 ± 1.6
3	25.8 ± 8.7	1.8 ± 0.9	1.9 ± 1.6	1.7 ± 1.3
4	15.1 ± 3.3	2.7 ± 0.4	2.8 ± 2.2	5.1 ± 3.5
Mean	24.2 ± 6.5	1.9 ± 0.6	3.4 ± 2.3	3.1 ± 1.9

Data are given as mean ± standard deviation

## Discussion

In this exploratory, proof-of-mechanism, *ex vivo* study, we have shown that a microbubble contrast agent can give contrast enhancement in clinically compatible GIM. Both the iodine and the microbubbles were visible in GIM, but detectable with two different image signals. This illustrates one of the key features of the GIM technique, namely, that it provides a set of three different images, each based on different physical properties, hence, potentially carrying different information: iodine is visible in the attenuation image because of its high attenuation coefficient while microbubbles become visible in the DF image due to its generation of ultra-small-angle scattering. Since microbubbles do not attenuate x-rays, the attenuation image can be used for anatomical orientation and the corresponding DF image for the assessment of contrast enhancement, which would result in a contrast-enhanced mammogram at a lower radiation dose and with a shorter acquisition time compared to conventional contrast-enhanced mammography [26]. Furthermore, microbubbles have different contrast kinetics compared to gadolinium and iodine than can be exploited in GIM. The assessment of wash-in and wash-out curves in the differentiation between benign and malignant lesions is a valuable tool in breast MRI, but the same has not been proven for iodine contrast-enhanced mammography [27]. As opposed to an early gadolinium wash-out signifying malignancy, microbubbles have been shown to have a persistent enhancement in malignant lesions [28], making it suitable for a bilateral mammography procedure. Finally, since microbubbles can be loaded with, *e.g.* drugs or genes, in combination with unloaded bubbles for imaging, there is a potential use also in theranostics [29, 30].

We found that the mean CNRs of the microbubbles were lower to that of iodine, which was expected especially considering that both contrast agents were used in its undiluted form. Iodine is normally diluted in clinical practice, whereas microbubbles are not. The main aim of this study was, however, only to determine whether there was a visible enhancement with the microbubbles in DF mode or not. Concerning test reliability, the experiment was repeated four times, and in all cases, the microbubbles were visible in the dark-field mode. However, there were several factors that led to a variation in the measurements such as the force applied to the contrast injection and a variation in sample thickness. Most importantly, the samples were not homogeneous leading to an uneven infiltration of the directly injected contrast agents.

If microbubbles are to be used as a contrast agent in GIM *in vivo*, further considerations have to be made concerning dose, and most importantly, the proper size of the bubbles. Theoretically, since the DF signal in grating interferometry is usually generated from small- or ultra-small-angle scattering, bubbles whose diameter matches the auto-correlation length of the setup could optimise the scattering signal [31, 32]. Consequently, the optimal bubble size for our investigational device would be 1 μm, *i.e.* slightly smaller than the commercial contrast agent actually used in the experiment.

To the best of our knowledge, this is the first time the feasibility of using microbubbles in a clinically compatible grating-interferometry phase-contrast imaging set-up has been shown. Velroy et al. [22] found that microbubbles scatter x-rays using grating-interferometry phase-contrast imaging in an experimental setting imaging vials with microbubbles with acquisition time and dose not optimised for clinical imaging. It is expected that the CNR of the DF contrast can be higher with a preclinical system compared with a clinical system, since the latter has constraints on geometry, radiation dose, and acquisition time. Without these constraints, the reduction of photon flux caused by the gratings can be compensated in the pre-clinical set-up to reduce the noise, by increasing the exposure time or making the interferometer shorter; in addition, since it is usually not subject to very strict geometric constraints, there is more freedom to optimise the geometry in terms of grating periods and inter-grating distances in order to have the most appropriate auto-correlation length.

There have been several studies on the DF signal as a function of the particle size [33–36]. For instance, Gkoumas et al. [36] measured colloidal suspensions of SiO_2_ microspheres of two different diameters (1.86 μm and 7.75 μm) in glycerine with increasingly higher concentrations (5–40%) on a synchrotron grating interferometer with an autocorrelation length of about 3.0 μm. They found that the utilised grating interferometer setup was more sensitive to higher concentrations. This outcome must be validated for our case, taking into account that we are measuring microbubble solution meant to be intravenously injected and that we are using a polychromatic setup.

However, even if we have demonstrated that microbubbles can provide visible contrast enhancement with a clinically compatible GIM device, the question whether this is a viable alternative *in vivo* remains to be answered and constitutes the main limitation of our study at this point. Additional studies on the appropriate bubble size as well as contrast agent concentration for GIM are needed.

In conclusion, GIM is a novel breast imaging technique at the edge of being clinically implemented. The method generates a set of three images based on the attenuation, refraction, and scattering of x-rays, which opens up the use of various contrast agents. This experiment set out to investigate whether microbubbles gave contrast enhancement *ex vivo* using a clinically compatible GIM setup. We found that microbubbles, due to their scattering properties, gave contrast enhancement in the dark-field mode. This implies the potential use of a contrast agent with a high safety profile, and with the prospect of using theranostics, in x-ray-based breast imaging.

## Abbreviations

CNR: Contrast-to-noise-ratio
DF: Dark-field
DPC: Differential phase contrast
GIM: Grating interferometry mammography
MRI: Magnetic resonance imaging
ROI: Region of interest
